# *In vivo* imaging of long-term accumulation of cancer-derived exosomes using a BRET-based reporter

**DOI:** 10.1038/s41598-020-73580-5

**Published:** 2020-10-06

**Authors:** Tomoya Hikita, Mamiko Miyata, Risayo Watanabe, Chitose Oneyama

**Affiliations:** 1 Division of Cancer Cell Regulation, Aichi Cancer Center Research Institute, Chikusa-ku, Nagoya, 464-8681 Japan; 2 Department of Target and Drug Discovery, Graduate School of Medicine, Nagoya University, Showa-ku, Nagoya, Japan; 3 Department of Oncology, Graduate School of Pharmaceutical Sciences, Nagoya City University, Mizuho-ku, Nagoya, Japan; 4 JST, PRESTO, Nagoya, Japan

**Keywords:** Cancer, Bioluminescence imaging

## Abstract

Monitoring of exosome dynamics in living organisms is essential to demonstrate the real functions of cancer-derived exosomes. Currently, these have been elucidated in vitro or under non-physiological conditions in vivo in most cases. To overcome these limitations, we developed an imaging method using Antares2-mediated bioluminescence resonance energy transfer (BRET) for observing long-term accumulation of exosomes in vivo. Ectopic expression of CD63-Antares2 effectively labeled exosomes with Antares2, which emitted intense, long-wavelength luminescence suitable for in vivo monitoring. Transplantation of CD63-Antares2-expressing prostate cancer cells into mice allowed determining the amount of cancer-derived exosomes released from primary tumors into the bloodstream and visualizing the long-term homing behavior of exosomes to their target organs or tissues. Interestingly, secreted exosome was decreased upon administration of low dose of dasatinib, an approved tyrosine-kinase inhibitor. The CD63-Antares2 xenograft mouse model will be useful for elucidating the dynamics of cancer-derived exosomes in vivo and evaluating the therapeutic efficacy and mechanism of exosome production inhibitors.

## Introduction

Exosomes are 50–150-nm-sized extracellular vesicles that mediate intercellular communication by transferring encapsulated biological molecules (e.g., nucleic acids, proteins, and lipids)^1^. Exosomes have various important physiological functions, such as stem cell maintenance^2,3^, tissue repair^4^, and immunosurveillance^5,6^,however, they also have pathological functions, especially in cancer, which have been intensively investigated. Several studies have shown that cancer-derived exosomes are related to cancer progression by promoting the development of a tumor microenvironment and/or premetastatic niche^7–11^. In most of these studies, isolated exosomes were added to cells or injected into animals, and the amount of exosomes used was not well considered^12^. To demonstrate the functions of cancer-derived exosomes in vivo*,* various imaging strategies have been developed to track them^13–16^. In these approaches, chemically or genetically labeled exosomes are injected directly into animal’s circulation, which allows monitoring only for a short period of time (less than a few days). Although these studies have provided important findings, it has not been confirmed whether the dose of exosomes administered and the route of administration are appropriate^17^. Further, exosomes prepared for exogenous injection may have a different heterogeneity from naturally secreted exosomes and contain other types of extracellular vesicles^18–20^. Therefore, it remains questionable whether the models used in these studies reflected the physiological dynamics of cancer-derived exosomes^12^. These situations highlight the need to develop a suitable in vivo imaging technique for monitoring the long-term distribution and accumulation of exosomes exuded from cancer cells.

As a first step to solve these problems, we replicated near-physiological conditions by developing a xenograft mouse model bearing tumor cells that express luminescent exosomes^21^. A thorough exosomal subclass analysis has demonstrated that tetraspanins CD63, CD81, and CD9 can be used as adequate markers of exosomes originated from late endosomes^22,23^. Thus, we have previously developed an ex vivo exosome-tracking method to monitor their long-term spatial behavior by labeling the exosome marker CD63 with high-intensity luciferase NanoLuc (Nluc)^21^. Although this method is effective for visualizing the long-term distribution of exosomes in tissues, it is not suitable for visualizing exosomes in vivo because the emission wavelength of Nluc (460 nm) is too short to penetrate mammalian tissues. To overcome this limitation, in this study, we employed the bioluminescence resonance energy transfer (BRET)-based reporter Antares2, which is a Nluc-based luciferase conjugated with CyOFP1, a cyan-excitable red fluorescent protein with an emission wavelength of 600 nm, as an acceptor of BRET. Ectopic expression of CD63-Antares2 effectively labeled exosomes with long-wavelength bioluminescence suitable for in vivo visualization.

## Results

### Detection of exosomes at long wavelength with Antares2-fused CD63

**Figure 1:**
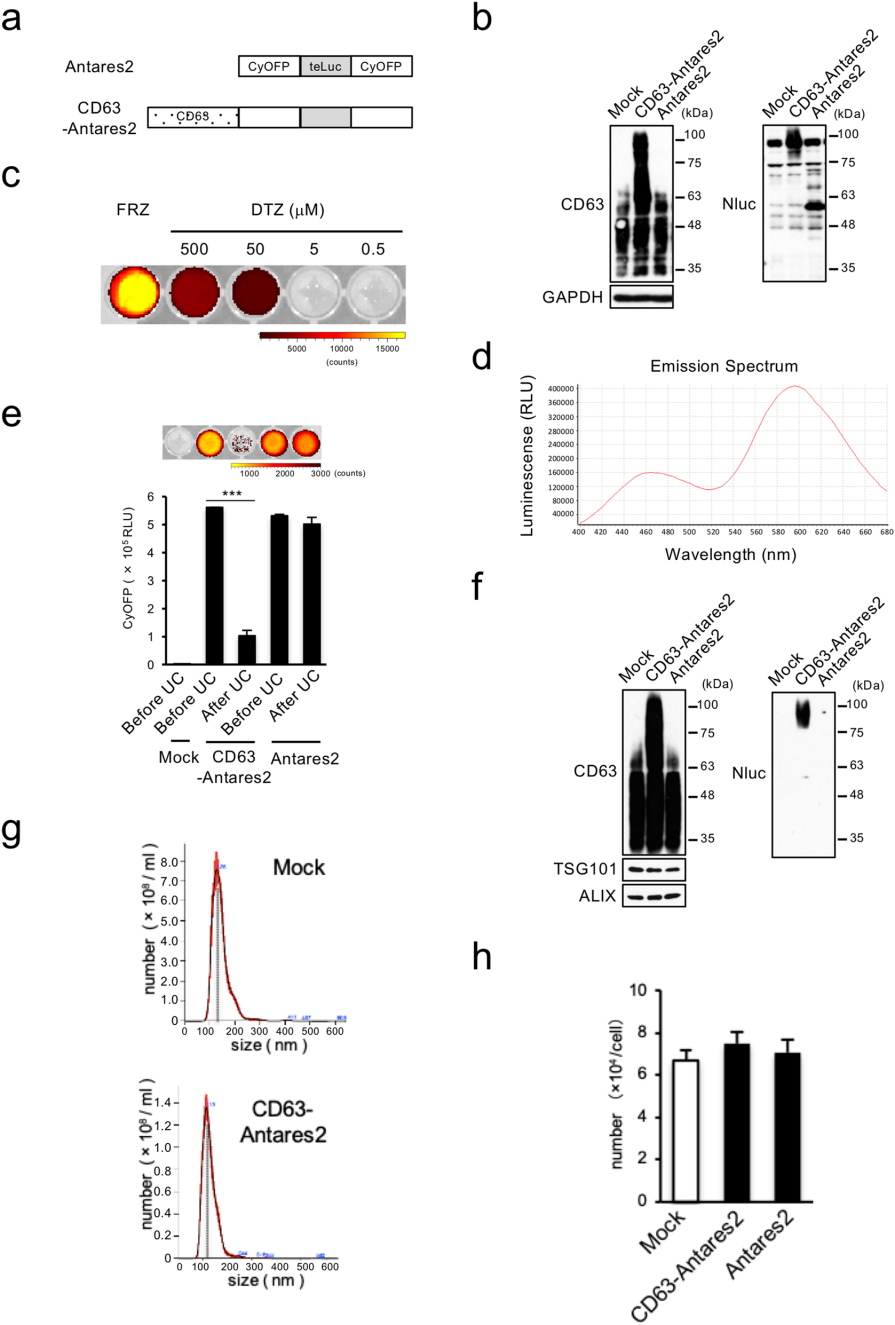
CD63-Antares2 expression enables the detection of exosomes at long wavelength. (**a**) Schematic diagram of Antares2 and Antares2-fused CD63 (CD63-Antares2). (**b**) Western blot analysis of control (Mock), CD63-Antares2-, and Antares2-expressing PC3 cells. Total cell lysates were immunoblotted with antibodies against the indicated proteins. (**c**) BRET signal of culture media containing PC3/CD63-Antares2 cells that were treated with FRZ or DTZ (500, 50, 5, and 0.5 μM) and imaged using the in vivo imaging system (IVIS). The data are representative of at least three independent experiments. (**d**) Emission spectrum of CD63-Antares2 in culture medium containing PC3/CD63-Antares2 cells. (**e**) BRET signal intensities in culture medium before and after ultracentrifugation (UC). The upper image shows BRET signals visualized using IVIS. (**f**) Western blot analysis of CD63-Antares2 and Antares2 expression in exosomes secreted from the cells indicated in (**b**). TSG101 and ALIX were used as exosome marker proteins. (**g**) Size distribution of isolated exosome particles derived from the cells indicated in (**b**) by NTA analysis. (**h**) Effect of ectopic CD63-Antares2 or Antares2 expression on exosome production. Exosome number per cell from the data (**g**) are expressed as means ± SD. All data are representative of at least 3 independent experiments each. ***P < 0.001, by ANOVA with Dunnett's post hoc analysis. Uncropped gel images for panels d and f are shown in Supplementary Fig. S3.

To develop a method to monitor cancer-derived exosomes, we used prostate cancer (PC3) cells as a model system because these cells secreted more exosomes than the other examined cell types (Supplementary Fig. S1a). We first transformed PC3 cells with a CD63-Akaluc construct as the near-infrared Akaluc/Akalumine system reportedly is optimal for deep-tissue imaging (Supplementary Fig. S1b)^24^. Although CD63-Akaluc-expressing PC3 cells produced intense luminescence and almost same of exosome number as that of parent cells (mock), exosomal luminescence secreted from cells was non-detectable in culture medium (Supplementary Fig. S1c–e). Therefore, we next evaluated the BRET system using the red-shifted reporter Antares2^25^, a Nluc mutant teLuc fused with CyOFP1 (Fig. 1a,b). A previous study reported that diphenylterazine (DTZ) was the optimal substrate for Antares2^26^,however, we found that furimazine (FRZ) produced a stronger signal than DTZ for detecting CD63-Antares2 in culture medium (Fig. 1c). Spectral analysis revealed that red-shifted luminescence (600 nm) of Antares2 was stronger than teLuc-derived signal (460 nm) in culture medium containing CD63-Antares2-expressing PC3 cells (Fig. 1d). To verify that the luminescence in culture medium was derived from exosomes, we compared the luminescence intensity before and after ultracentrifugation and quantified the CD63-Antares2 protein in isolated exosomes. The luminescence intensity of culture medium of CD63-Antares2-expressing PC3 cells was drastically reduced by ultracentrifugation, while it was not changed in the case of Antares2-expressing cells (Fig. 1e). And CD63-Antares2 was detected only in exosomes derived from CD63-Antares2-expressing cells (Fig. 1f). Thus, we concluded that CD63-fused Antares2 was mostly contained into secreted exosomes. Exosomes derived from CD63-Antares2-expressing cells and their parent cells showed nearly the same size and number (Fig. 1g,h). Antares2-derived bioluminescence intensity in culture medium was closely correlated with the numbers of cells and exosomes (Supplementary Fig. S1f,g). These findings suggest that labeling exosomes with CD63-Antares2 is suitable for their tracking in vivo.

### Exosome tracking analysis in vivo by using Antares2-labeled exosome-producing cells

**Figure 2:**
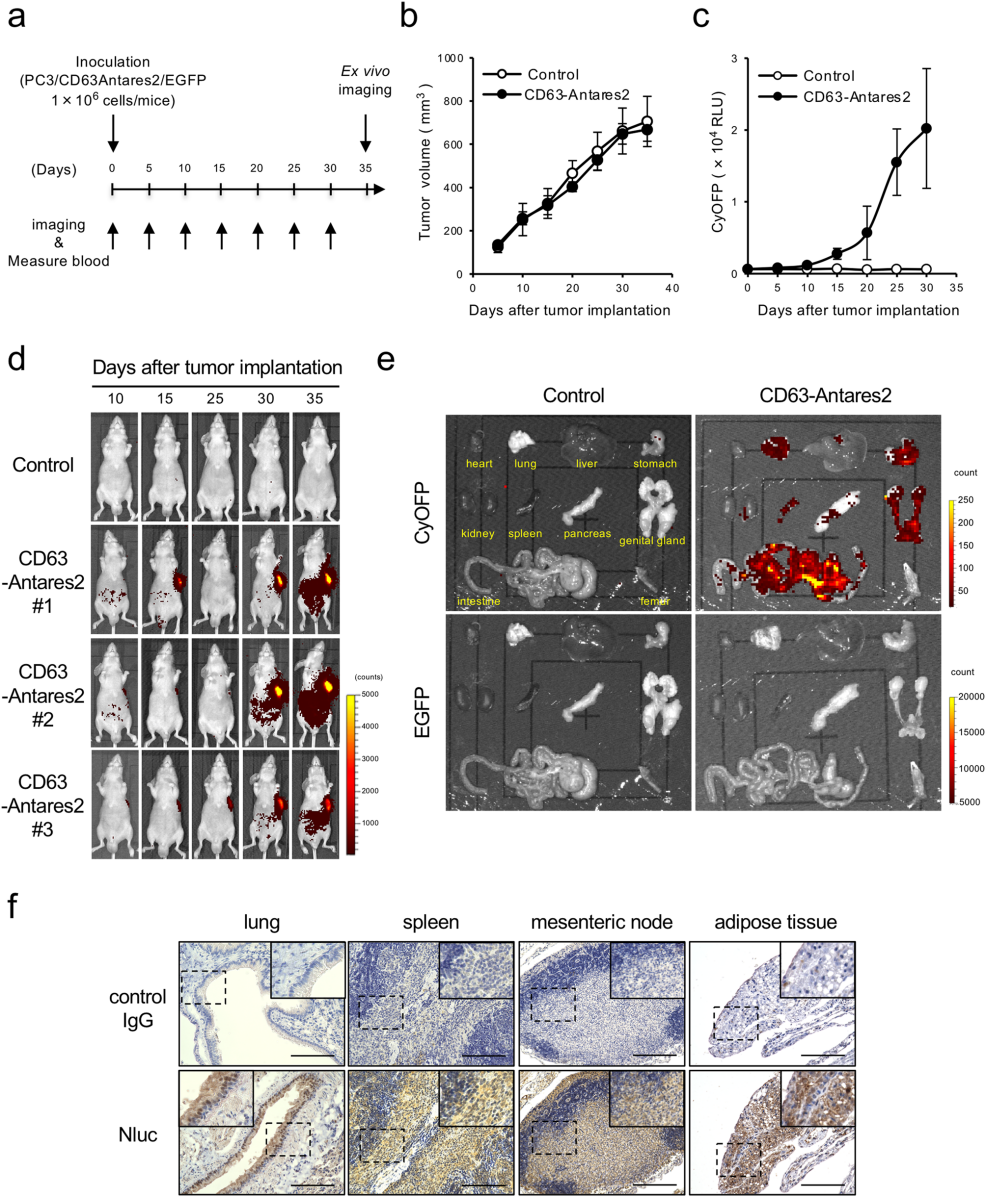
PC3/CD63-Antares2 xenograft mice model enables continuous exosome tracking analysis in vivo. (**a**) Timeline of in vivo exosome tracking analysis in a xenograft mouse model established using PC3/CD63-Antares2- and EGFP-expressing (PC3/CD63-Antares2/EGFP) cells. Whole body imaging analyses using IVIS and BRET intensity measurement in blood samples were performed every 5 days after subcutaneous inoculation of control (PC3/Mock) or PC3/CD63-Antares2/EGFP cells into the mice. (**b**) Tumor growth in control (PC3/Mock) and PC3/CD63-Antares2/EGFP xenograft mice. (**c**) Time course of BRET signal in the blood of control (PC3/Mock) and PC3/CD63-Antares2/EGFP xenograft mice. (**d**) Sequential bioluminescence images of the whole body of control (PC3/Mock) and PC3/CD63-Antares2/EGFP xenograft mice. BRET emission derived from Antares2 was imaged using IVIS. (**e**) Bioluminescence images of organs harvested from control (PC3/Mock) and PC3/CD63-Antares2/EGFP tumor-bearing mice 5 weeks after inoculation. BRET emission derived from Antares2 and EGFP fluorescence were imaged using IVIS. (**f**) Immunohistochemical analysis of Nluc (Antares2) in the lungs, spleen, mesenteric nodes, and adipose tissues from mice bearing PC3/CD63-Antares2/EGFP cells (40 ×). Boxed with dotted line images are enlarged inset of the panels. Scale bar = 100 μm. Representative images from three mice are shown in (d, e, and f). Means ± SDs of tumor volume and luminescence intensity of BRET were obtained from three mice (b and c).

We next attempted to visualize the biodistribution of circulating cancer-derived exosomes by subcutaneously transplanting PC3/CD63-Antares2 cells into nude mice. To monitor circulating and homing cancer-derived exosomes, we continuously measured luminescence intensity in the blood and performed whole-body bioluminescence imaging for 35 days (Fig. 2a). Under experimental conditions in which control and CD63-Antares2-expressing cells showed the same tumor growth (Fig. 2b), the bioluminescence intensity in the blood gradually increased with increasing PC3/CD63-Antares2 tumor size (Fig. 2c). Whole-body imaging revealed the appearance of the reporter signal in PC3/CD63-Antares2 tumor-bearing mice accompanied with an increase in bioluminescence intensity in the blood (Fig. 2d). We next investigated homing tissues of PC3-derived exosomes using excised organs. We detected bioluminescence signals in various organs, including the lungs, stomach, intestine, and genital glands (Fig. 2e, upper panels). To distinguish between exosome-derived and cell-derived luminescence, we labeled the PC3/CD63-Antares2 cells with EGFP. The results confirmed that cell-derived EGFP signal was almost not detected in these organs, although EGFP-expressing PC3/CD63-Antares2 cells showed luminescent signal in cell-number dependent manner (Fig. 2e, lower panels and Supplementary Fig. S1h). Further, to verify the cellular uptake of exosomes, we performed immunohistological analysis using an anti-Nluc antibody crossed with Antares2. We observed Nluc signal in bronchoepithelial cells in the lungs, lymphocyte and erythrocyte in the spleen, mesenteric lymph nodes, and adipocyte in the adipose tissues in the genital glands and intestine (Fig. 2f). Taken together, these results indicated that the xenograft mouse model bearing CD63-Antares2-expressing cancer cells allows monitoring long-term circulating cancer-derived exosomes quantitatively and in a time-dependent manner, and visualizing exosome-homing organs, tissues, and cells.

### Application of Antares2-labeled exosome-producing cells to evaluate exosome secretion inhibitor

**Figure 3:**
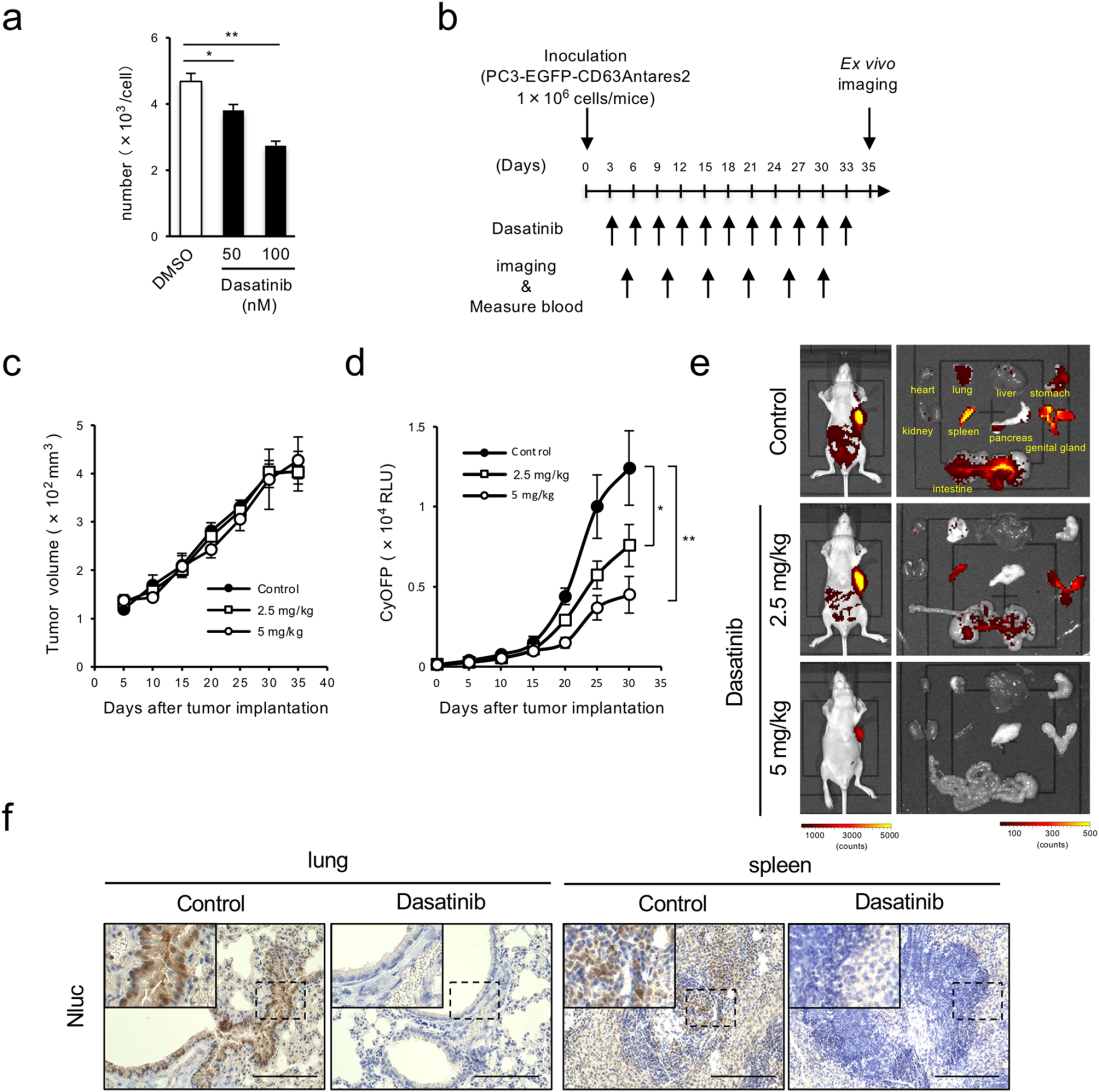
The PC3/CD63-Antares2 xenograft mouse model is useful for evaluating the effects of exosome inhibitors in vivo. (**a**) Numbers of exosomes produced by PC3/CD63-Antares2 cells treated with DMSO or dasatinib (50 or 100 nM). (**b**) Timeline of in vivo exosome tracking analysis using the PC3/CD63-Antares2 xenograft mouse model. Dasatinib was administrated intraperitoneally once every 3 days. Whole body imaging using IVIS and BRET intensity measurement using blood samples were performed every 5 days after subcutaneous inoculation of PC3/CD63-Antares2 cells into the mice. (**c**) PC3/CD63-Antares2 cells were inoculated subcutaneously into the mice. When the tumor volume reached approximately 100 mm^3^, saline (Control) or dasatinib (2.5 mg/kg or 5 mg/kg) was administrated. Mean ± SD values of tumor volume (mm^3^) obtained from 3 mice are plotted against days after inoculation. (**d**) Sequential BRET signal in the blood samples from control (saline administrated) or dasatinib-administrated (2.5 mg or 5 mg/kg) PC3/CD63-Antares2 xenograft mice. Means ± SDs of BRET signal obtained from three mice are plotted against days after inoculation. (**e**) Bioluminescence images of organs excised from control (saline-administrated) and dasatinib-administrated PC3/CD63-Antares2 xenograft mice 5 weeks after inoculation. (f) Immunohistochemical analysis of Nluc (Antares2) in the lungs and spleen from control (saline administrated) and dasatinib-administrated PC3/CD63-Antares2 xenograft mice (40 ×). Boxed in dotted line images are enlarged inset of the panels. Scale bar = 100 μm. *P < 0.05, **P < 0.01, by ANOVA with Dunnett's post hoc analysis.

Furthermore, we examined whether this system could be applied for evaluating exosome inhibitors as potential new cancer therapeutics^27,28^. We examined the effects on bioluminescence of a well-known exosome secretion inhibitor, GW4869, and an promoter of exosome production, bafilomycin A1 by adding these chemicals to the culture medium of PC3/CD63-Antares2 cells^29,30^ (Supplementary Fig. S2a,b). Under these conditions, bioluminescence in the culture medium increased by bafilomycin A1, although it was not changed by GW4869. In addition, as we previously found that the non-receptor tyrosine kinase Src promotes the secretion of cancer-derived exosomes and the broad tyrosine kinase inhibitor dasatinib effectively suppresses exosome secretion^31^. We treated the cells with several mutikinase inhibitors including dasatinib, SU6656, saracatinib, ponatinib, or bosutinib for 24 h. Each inhibitor decreased cellular tyrosine phosphorylation at different concentration and decreased the luminescence intensity in culture medium containing PC3/CD63-Antares2 cells, associated with the suppression of Src activity (Supplementary Fig. S2c,d). Dasatinib, ponatinib, and bosutinib significantly reduced the luminescence intensity in culture medium from these cells (Supplementary Fig. S2d). Consistent herewith, dasatinib, ponatinib, and bosutinib suppressed exosome secretion from these cells (Fig. 3a and Supplementary Fig. S2e). The luminescence intensity and exosome number were suppressed in dose-dependent manner of dasatinib, accompanied with Src inhibition (Supplementary Fig. S2c,f). Since dasatinib suppressed exosome secretion at lower concentration than ponatinib and bosutinib, we then examined the inhibitory effect of intraperitoneally injected dasatinib in the PC3/CD63-Antares2 xenograft model mice by measuring the luminescence intensity under the same conditions as those used to produce the data shown in Fig. 2a (Fig. 3b). Administration of dasatinib at a low dose (5 mg/kg) had no effect on tumor growth (Fig. 3c and Supplementary Fig. S2g), but it drastically decreased the luminescence intensity of CD63-Antares2 cells in both the blood and homing organs in a dose-dependent manner (Fig. 3d,e). We confirmed that dasatinib did not affect stability of exosomes secreted from cancer cells (Supplementary Fig. S2h). To evaluate the effect of dasatinib on the homing of exosomes derived from PC3/CD63-Antares2 cells in more detail, we subjected excised organs to immunohistochemical analysis. While Nluc luminescence was intensely observed in both the lungs and spleen from control mice, it was drastically weakened in these tissues obtained from mice administered dasatinib (Fig. 3f). Taken together, these results suggested that the xenograft mouse model bearing CD63-Antares2-expressing cancer cells is suitable for in vivo evaluation of drugs designed to inhibit exosome secretion.

## Discussion

To achieve the sensitivity required for imaging exosomes in deep tissues and organs, we first used Akaluc, which was derived from firefly luciferase, as a near-infrared reporter; however, luminescence of Akaluc-labeled exosomes secreted from cells could not be successfully detected in culture medium. This may be due to the physical properties of Akaluc, such as ATP dependency and pH sensitivity outside the neutral range^24^. While the actual pH of secreted exosomes has not yet been reported, their internal environment may be acidic, because they are formed as intraluminal vesicles in multivesicular endosomes matured from endosomes^32^, leading to Akaluc inactivation. Therefore, reporters derived from pH-labile firefly luciferase would not be suitable for detecting exosomes. On the contrary, the Nluc-derived reporter exhibited high sensitivity for exosome detection, whereas the signal intensity of Nluc was insufficient for noninvasive exosome imaging as previously demonstrated^21^. Here, we have achieved in vivo exosome imaging for a month by using the CD63-conjugated BRET-based red-shifted reporter Antares2 and FRZ as its substrate.

Organotropic metastasis of cancer cells reportedly depends on integrins on exosomes^11^, however, the molecular mechanisms underlying organotropism of exosomes remain elusive. By using CD63-Nluc-labeled exosomes, we previously demonstrated that HT29 colon cancer cell-derived exosomes were preferentially recruited in the stomach and intestine after 7 weeks, and Src-transformed mouse embryonic fibroblast-derived exosomes accumulated in the lungs^21^. The CD63-Antares2 xenograft mouse model might be useful for analyzing organotropism of cancer-derived exosomes in living animals, in a time-dependent manner. In the PC3/CD63-Antares2 xenograft model mice, PC3 cancer cell-derived exosomes selectively accumulated in the lungs, spleen, lymph nodes, and adipose tissues after long-term circulation. In contrast to these findings, previous studies of short-term distribution after exosome injection have shown that exosomes mostly accumulated in the liver and lungs^16^. Recently, exosomes have been shown to function in immune responses in organs that activate immune cells, such as the spleen and lymph nodes^33,34^. Notably, PC3-derived exosomes highly express programmed cell death ligand 1, which promotes T-cell exhaustion in draining lymph nodes^35–38^. Although we could not analyze the function of PC3 cell-derived exosomes in nude mice with defect of immune system bearing PC3/CD63-Antares2 tumors, further analysis of their effects on immune cells in the spleen and lymph nodes would be valuable.

Given the importance of cancer-derived exosomes in cancer progression, drug discovery targeting exosomes has been vigorously pursued over the past years^27,28^. We demonstrated that in CD63-Antares2 xenograft model mice, dasatinib at 2.5 mg/kg inhibited exosome secretion without suppressing tumor growth. Exosomes derived from targeted CD63-Antares2-expressing cancer cells could be a promising tool for evaluating the efficacy of exosome inhibitors in vivo.

In summary, we demonstrated that the CD63-Antares2 xenograft mouse model represents a powerful tool for in vivo imaging of the long-term distribution of exosomes. This model could be applied for investigating the function of cancer-derived exosomes in homing tissues, mechanisms of organotropism, and discovery of drugs targeting cancer-derived exosomes, thereby contributing to the overall development of exosome biology.

## Methods

### Ethics approval

All animal experiments were performed under protocols approved by the Animal Care and Use Committee of Aichi Cancer Center Research Institute (No. 31–17). All analyses were performed in accordance with approved guidelines and regulations.

### Cell culture and reagents

HT29 (ATCC HTB-38) and HCT116 (ATCC CCL-247) cells were cultured in McCoy’s 5A medium (Gibco, Gaithersburg, MD, USA) supplemented with 10% fetal bovine serum (FBS; Gibco), 100 U/mL penicillin G, and 100 μg/mL streptomycin at 37 °C in the presence of 5% CO_2_. MDA-MB-231 (ATCC HTB-26), A549 (ATCC CCL-185), PANC-1 (ATCC CRL-1469), and MIAPaCa-2 (ATCC CRL-1420) cells were cultured in Dulbecco’s modified Eagle’s medium (Sigma-Aldrich, St. Louis, MO, USA) supplemented with 10% FBS, 100 U/mL penicillin G, and 100 μg/mL streptomycin at 37 °C in the presence of 5% CO_2_. DU145 (RCB 2143), LNCaP (RCB 2144), and PC3 (RCB 2145) cells were purchased from RIKEN RBC and were grown in RPMI-1640 (Sigma-Aldrich, St Louis, MO, USA) supplemented with 10% FBS, 100 U/mL penicillin G, and 100 μg/mL streptomycin at 37 °C in the presence of 5% CO_2_. Dasatinib (S1021) was purchased from Selleck (Houston, TX, USA) and GW4869 (D1692) was obtained from Sigma-Aldrich. Bafilomycin A1 (BVT-0252) was purchased from BioViotica (Dransfeld, Germany).

### Vector construction

All gene transfer experiments were conducted using the pCX4 series of retroviral vectors. Nluc, Antares2, and Akaluc amplicons, amplified from pNLF1-C (Promega, Madison, WI, USA), pcDNA3-Antares2 c-myc (Addgene #100,027, deposited by Huiwang Ai), and pcDNA3 Venus-Akaluc (RDB15781; RIKEN BRC), were respectively subcloned into pCX4bsr. pCX4bsr-CD63Nluc, pCX4bsr-CD63Antares2, and pCX4bsr-CD63Akaluc expression vectors were generated by cloning a human CD63 amplicon into the reporter-modified vectors. EGFP amplicon, amplified from pEGFP-N1 (Clontech Laboratories, Mountain view, CA, USA), was subcloned into pCX4bleo. The retroviral vectors were produced and transfected into cells as described previously^39–41^.

### Western blotting

Cells and exosomes were lysed in n-octyl-β-d-glucoside buffer (20 mM Tris–HCl, pH 7.4, 150 mM NaCl, 1 mM EDTA, 1 mM sodium orthovanadate, 20 mM NaF, 1% Nonidet P-40, 5% glycerol, 2% n-octyl-β-d-glucoside, and protease inhibitor cocktail), and immunoblotting was performed as described previously^40^. The following antibodies were used: anti-Alix (ABC40, Merck, Darmstadt, Germany), anti-TSG101 (C-2, Santa Cruz, Santa Cruz, CA, USA), anti-CD63 (MX-49.129.5, Santa Cruz), anti-phospho-tyrosine (pY,4G10, Merck), anti-Src pY416 (D49G4, Cell Signaling Technology, Danvers, MA, USA), anti-Src (Ab-1, Merck), and anti-GAPDH (6C5, Santa Cruz). Anti-Nluc rabbit polyclonal antibody was kindly provided by Promega (Madison, WI, USA).

### Exosome preparation and nanoparticle tracking analysis

CD63-Antares2-expressing PC3 (PC3/CD63-Antares2) cells were seeded on a 150-mm culture dish at a density of 5 × 10^3^ cells and cultured for 24 h. After washing with 20 mL of PBS twice, the culture medium was replaced with 13 mL of medium containing 1% exosome-depleted FBS. After 48 h of culture, to remove cells and cellular debris, the supernatant was centrifuged at 2 000 × *g* at 4 °C for 10 min and then filtered through a 0.22-μm filter (Merck). To prevent exosome aggregation, trehalose (1 M trehalose in 20 mM HEPES, pH 7.4) was added to the filtered supernatant at a final concentration of 25 mM^42^. The supernatants were ultracentrifuged at 110 000 × *g* at 4 °C (SW41Ti rotor, Beckman Coulter, Brea, CA, USA) for 70 min and the pellets were washed with 11 mL of trehalose-containing HEPES buffer (20 mM HEPES pH 7.4, 25 mM trehalose). The suspensions were re-ultracentrifuged at 110 000 × *g* at 4 °C for 70 min and finally suspended in 200 μL HEPES buffer (20 mM HEPES, pH 7.4)^26^. The size distribution and concentration of the exosomes were determined by nanoparticle-tracking analysis using a NanoSight LM10 instrument (Malvern, Worcestershire, UK) equipped with a 488-nm laser and NTA3.1 software. Five individual 30-s measurements were recorded for each sample using automated analysis settings for blur, track length, and minimum expected particle size. The camera level was set at 14 and the detection threshold at 10 in NTA3.1 software.

### In vivo exosome tracking analysis

A mixture of EGFP-labeled PC3/CD63-Antares2 cells (1 × 10^6^ cells/100 μL) and Matrigel (100 μL) was subcutaneously implanted into 5-week-old BALB/c-nu/nu male mice (Japan SLC Inc., Shizuoka, Japan). Seven weeks later, 100 μL of Nano-Glo reagent diluted at 1:20 (v/v) in sterile phosphate-buffered saline (PBS) was intraperitoneally injected into the mice. Three minutes after this injection, the mice were euthanized by cervical dislocation and the organs were harvested within 7 min as previously reported^21^. For drug administration into the mice, saline (Control) or dasatinib (2.5 mg/kg or 5 mg/kg) was administrated, when the tumor volume reached approximately 100 mm^3^. Luminescence intensity in the isolated organs was quantified using an IVIS Lumina II imaging system (PerkinElmer, Waltham, MA, USA).

### Luciferase assay

Cells and cellular debris were removed from the culture medium by centrifugation at 2 000 × *g* at 4 °C for 10 min, and 50 μL of the supernatant was transferred to white-walled 96-well plates. Nano-Glo substrate (50 μL; FRZ) diluted at 1:50 (v/v) in the buffer provided with the Nano-Glo Luciferase Assay System (Promega) was added to the plates and the luciferase intensity was measured immediately using a VICTOR Nivo Multiplate Reader (PerkinElmer). For spectral scanning, a CLARIOstar plate reader (BMG Labtech, Aylesbury, UK) was used. To assess DTZ, another substrate of Antares2, 50 μL of DTZ (HY-111382; MedChemExpress, Monmouth Junction, NJ, USA) at the indicated concentrations was added to 50 μL of supernatant. To assess Akaluc, Akalumine-HCl (150 μM; FUJIFILM Wako chemicals, Osaka, Japan) was added to 50 μL of supernatant.

### Immunohistochemical analysis

The harvested mouse organs were fixed in 4% (v/v) paraformaldehyde. Briefly, paraffin-embedded section slides were deparaffinized and treated with citrate buffer, pH 6.0, at 120 °C for 5 min. After washing with PBS, endogenous peroxidase was blocked with 0.3% H_2_O_2_ for 30 min. The slides were blocked with Blocking One Histo (Nacalai, Kyoto, japan) for 30 min and then incubated with primary antibodies or IgG isotype control overnight at 4 °C. After washing twice with PBS, the slides were incubated with Envision + Dual Link System-HRP (Dako, Santa Clara, CA, USA) for 30 min. The slides were developed using the Peroxidase Stain DAB Kit (Nacalai), a chromogenic substrate for peroxidase, and counterstained with hematoxylin. The slides were visualized and imaged under a fluorescence microscope (BZ-X710; Keyence, Osaka, Japan).

### Statistical analysis

All summary data were reported as the means ± S.D. calculated for each group and compared using ANOVA followed by Dunnett's post hoc test. Test results were reported as two-tailed *p* values, where *p* < 0.05 was considered statistically significant.

### Data availability

All data generated or analyzed during this study are included in this published article.

## Supplementary information

Supplementary Information.
