# Supplementary material for: In vivo imaging of long-term accumulation of cancer-derived exosomes using a BRET-based reporter
Source: Sci Rep. 2020 Oct 6;10:16616. doi: 10.1038/s41598-020-73580-5 (PMC7538576; doi:10.1038/s41598-020-73580-5)
Supplement: Supplementary file 1 — Supplementary Information. [file 41598_2020_73580_MOESM1_ESM.pdf]

Supplementary Information

In vivo imaging of long-term accumulated cancer-derived exosomes using a BRET-based reporter

Tomoya Hikita[1], Mamiko Miyata[1], Risayo Watanabe[1], and Chitose Oneyama[1, 2, 3, 4]*

[1] Division of Cancer Cell Regulation, Aichi Cancer Center Research Institute, Chikusa-ku, Nagoya, Japan

[2] Department of Target and Drug Discovery, Nagoya University, Graduate School of Medicine, Showa-ku, Nagoya, Japan

[3] Department of Oncology, Nagoya City University, Graduate School of Pharmaceutical Sciences, Mizuho-ku, Nagoya, Japan

[4] JST, PRESTO, Nagoya, Japan

*Correspondence should be addressed to: Chitose Oneyama, Ph.D.

Division of Cancer Cell Regulation, Aichi Cancer Center Research Institute, Chikusa-ku, Nagoya 464-8681, Japan

E-mail: coneyama@aichi-cc.jp

Tel: +81-52-764-2979

Fax: +81-52-763-5233

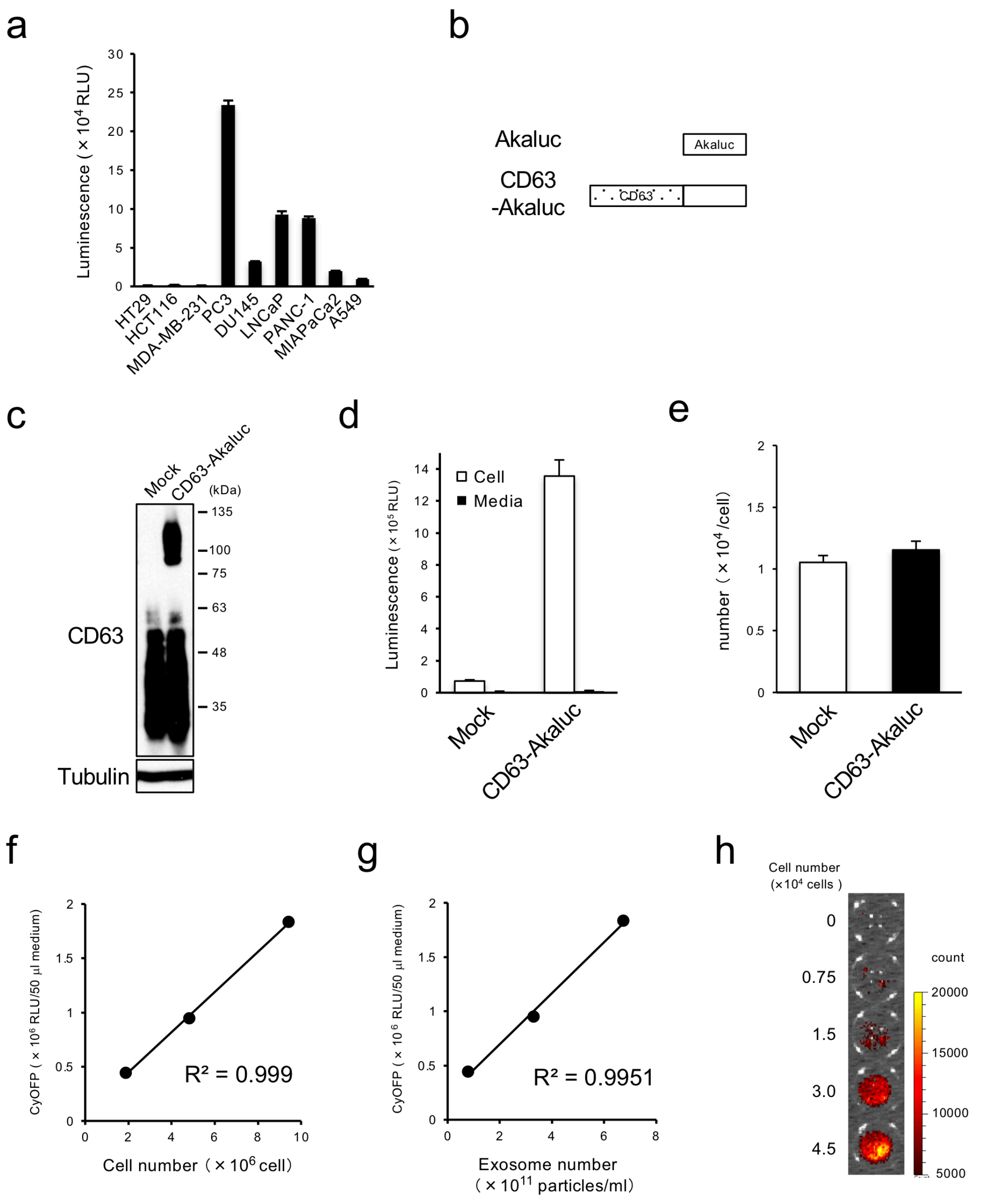

Figure S1

(a) NanoLuc luciferase intensity in the culture medium of various Nluc-fused CD63 (CD63-Nluc)-expressing cancer cell types. (b) Schematic diagram of Akaluc and Akaluc-fused CD63 (CD63-Akaluc). (c) Western blot analysis of CD63 expression in mock-infected control PC3 cells (Mock) and CD63-Akaluc expressing PC3 cells. Total cell lysate was immunoblotted with antibodies against the indicated proteins. (d)

Akaluc-derived luminescence in cells (white bar) or in the culture medium (black bar). (e) Numbers of exosomes produced by PC3/CD63-Akaluc cells as indicated in (c). (f) Correlation between BRET signal (in culture medium) and seeded cell number. The solid line shows the linearity of the fitted curve of luminescence vs. seeded cell number. (g) Correlation between BRET signal (in culture medium) and exosome number. The solid line shows the linearity of the fitted curve of luminescence vs. exosome number. (h) BRET signal of culture media containing EGFP-expressing PC3/CD63-Antares2 cells was imaged using the *in vivo* imaging system (IVIS). The data are representative of at least three independent experiments. For all graphs, the error bars indicate the mean ± SD of three independent experiments.

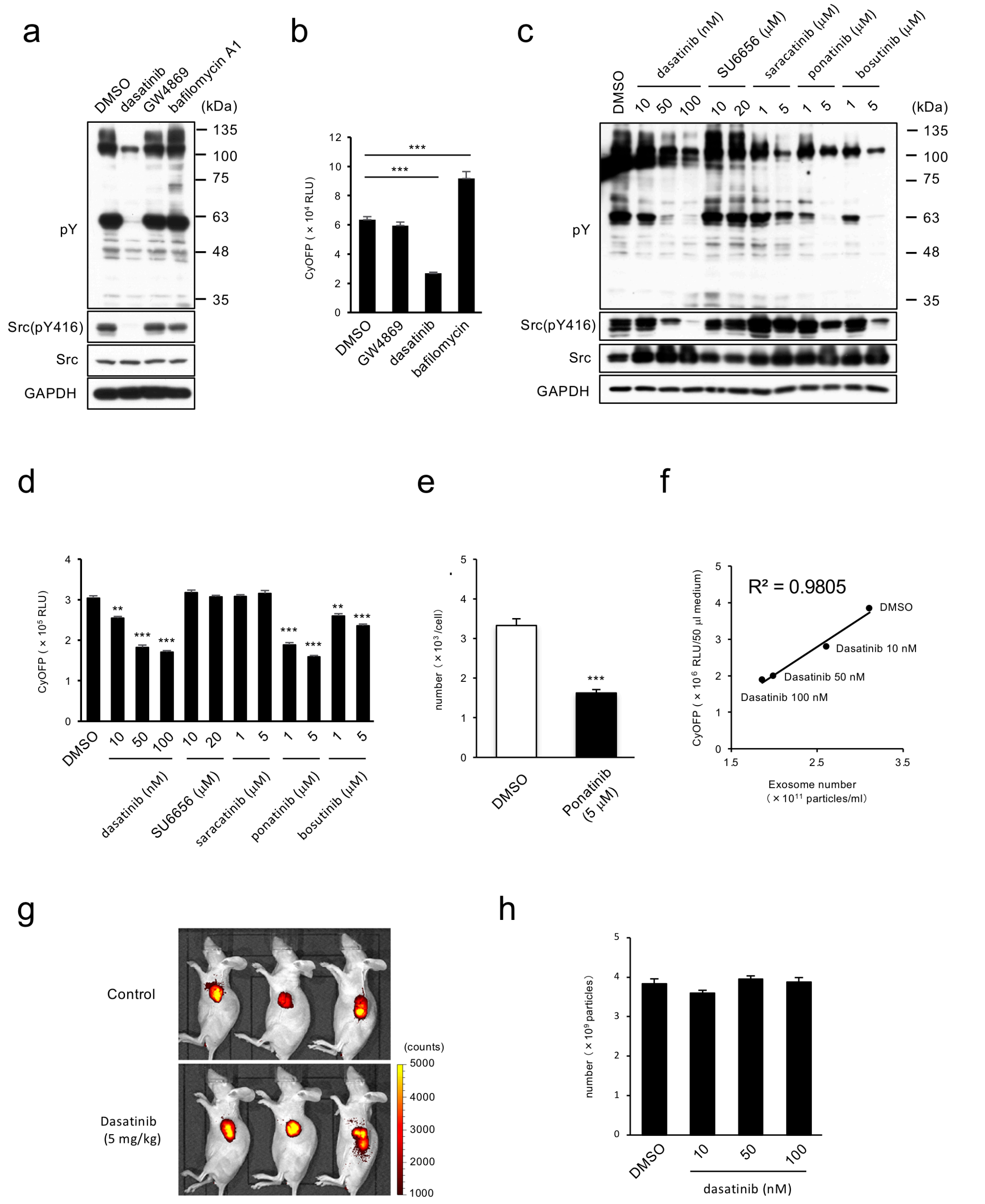

Figure S2

(a) Western blot analysis of PC3/CD63-Antares2 cells treated with DMSO (as a control), 100 nM dasatinib, 10 μM GW4869, 50 nM bafilomycin A1. Total cell lysates were immunoblotted with antibodies against the indicated proteins (pY: phophotyrotsine,

Src(pY416): activated Src). (b) BRET signal in the culture medium of PC3/CD63-Antares2 cells treated with the indicated chemicals. PC3/CD63-Antares2 cells were treated with GW4869 (10 μM), dasatinib (100 nM), or bafilomycin A1 (50 nM). (c) Western blot analysis of PC3/CD63-Antares2 cells treated with DMSO (as a control), dasatinib, SU6656, saracatinib, ponatinib, or bosutinib at different concentrations. Total cell lysates were immunoblotted with antibodies against the indicated proteins (pY: phophotyrotsine, Src(pY416): activated Src). (d) BRET signal in the culture medium of PC3/CD63-Antares2 cells treated with the indicated chemicals used in (c). (e) Numbers of exosomes secreted by PC3/CD63-Antares2 cells treated with DMSO or 50 μM dasatinib. (f) Correlation between BRET signal (in culture medium) and exosome number from the cells treated with indicated concentration of dasatinib. The solid line shows the linearity of the fitted curve of luminescence vs. exosome number. (g) Bioluminescence images of dasatinib-administrated PC3/CD63-Antares2 xenograft mice 5 weeks after inoculation. (h) Effect of dasatinib on the stability of secreted exosomes. Exosomes secreted from PC3/CD63-Antares2 cells were incubated with DMSO (as a control), or different concentration of dasatinib at 37˚C for 3 hours and were subjected to NTA analysis. Exosome number are expressed as means ± SD. For all graphs, the error bars indicate the mean ± SD of three independent experiments. $^{**}P < 0.01$, $^{***}P < 0.001$, by ANOVA with Dunnett's post hoc analysis.

Figure 1 b

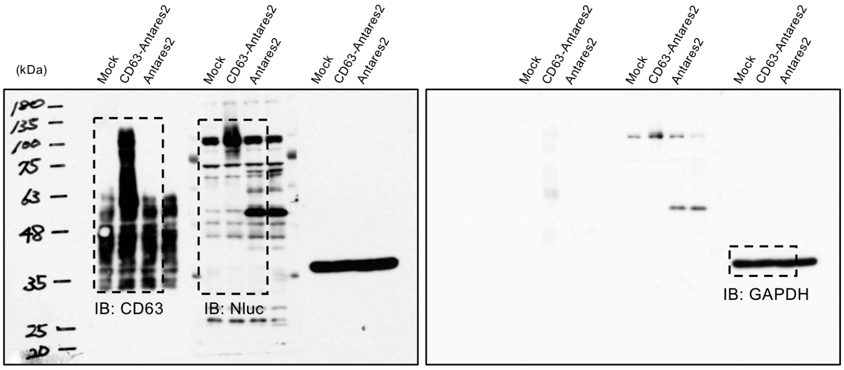

Figure 1 f

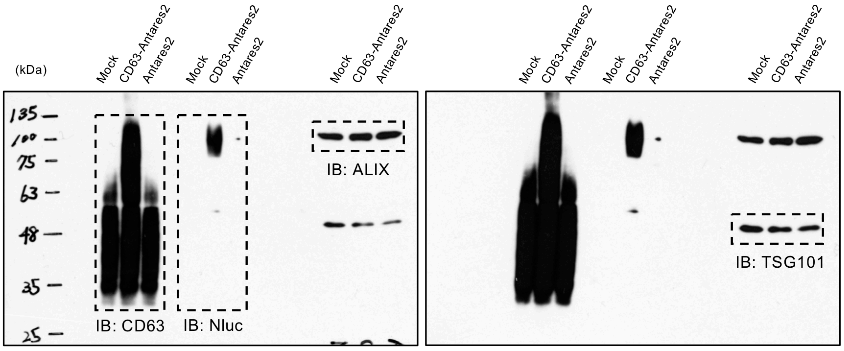

SupFigure 1 b

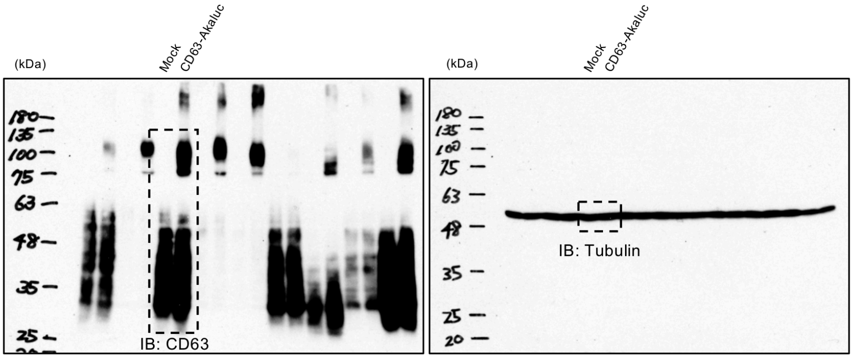

SupFigure 2 a

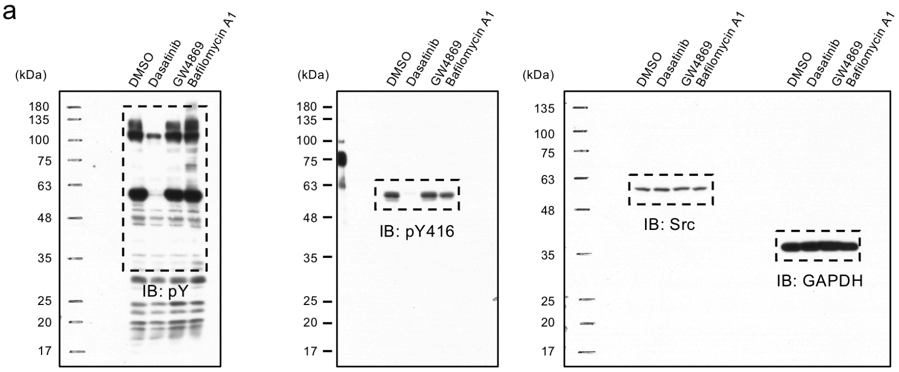

SupFigure 2 c

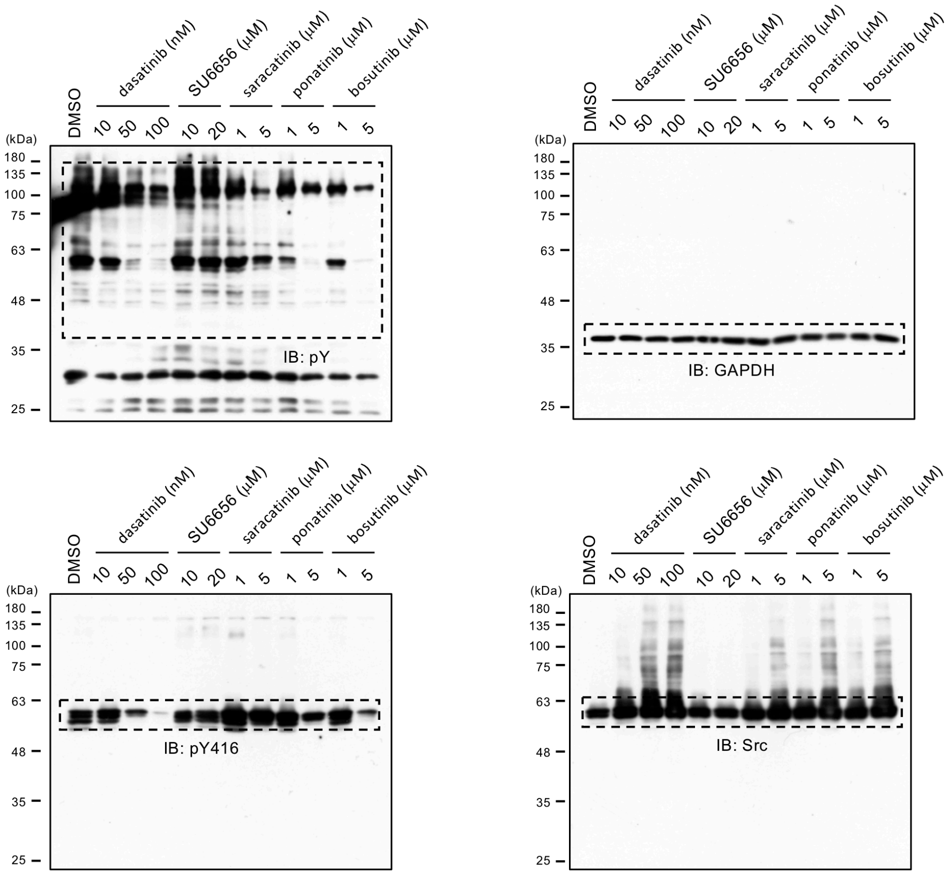

Figure S3

Full scan images of Figures.
